# Assessment of genetic variation within a global collection of lentil (*Lens culinaris* Medik.) cultivars and landraces using SNP markers

**DOI:** 10.1186/s12863-014-0150-3

**Published:** 2014-12-24

**Authors:** Maria Lombardi, Michael Materne, Noel O I Cogan, Matthew Rodda, Hans D Daetwyler, Anthony T Slater, John W Forster, Sukhjiwan Kaur

**Affiliations:** Department of Environment and Primary Industries, Biosciences Research Division, AgriBio, Centre for AgriBioscience, La Trobe University, 5 Ring Road, Bundoora, Melbourne, 3083 Victoria Australia; Department of Environment and Primary Industries, Biosciences Research Division, Grains Innovation Park, Horsham, 3401 Victoria Australia; La Trobe University, Bundoora, Melbourne, 3086 Victoria Australia

**Keywords:** Grain legume, Genetic diversity, Genotyping, Principal coordinate analysis, Dendrogram, Plant breeding

## Abstract

**Background:**

Lentil is a self-pollinated annual diploid (2n = 2× = 14) crop with a restricted history of genetic improvement through breeding, particularly when compared to cereal crops. This limited breeding has probably contributed to the narrow genetic base of local cultivars, and a corresponding potential to continue yield increases and stability. Therefore, knowledge of genetic variation and relationships between populations is important for understanding of available genetic variability and its potential for use in breeding programs. Single nucleotide polymorphism (SNP) markers provide a method for rapid automated genotyping and subsequent data analysis over large numbers of samples, allowing assessment of genetic relationships between genotypes.

**Results:**

In order to investigate levels of genetic diversity within lentil germplasm, 505 cultivars and landraces were genotyped with 384 genome-wide distributed SNP markers, of which 266 (69.2%) obtained successful amplification and detected polymorphisms. Gene diversity and PIC values varied between 0.108-0.5 and 0.102-0.375, with averages of 0.419 and 0.328, respectively. On the basis of clarity and interest to lentil breeders, the genetic structure of the germplasm collection was analysed separately for cultivars and landraces. A neighbour-joining (NJ) dendrogram was constructed for commercial cultivars, in which lentil cultivars were sorted into three major groups (G-I, G-II and G-III). These results were further supported by principal coordinate analysis (PCoA) and STRUCTURE, from which three clear clusters were defined based on differences in geographical location. In the case of landraces, a weak correlation between geographical origin and genetic relationships was observed. The landraces from the Mediterranean region, predominantly Greece and Turkey, revealed very high levels of genetic diversity.

**Conclusions:**

Lentil cultivars revealed clear clustering based on geographical origin, but much more limited correlation between geographic origin and genetic diversity was observed for landraces. These results suggest that selection of divergent parental genotypes for breeding should be made actively on the basis of systematic assessment of genetic distance between genotypes, rather than passively based on geographical distance.

**Electronic supplementary material:**

The online version of this article (doi:10.1186/s12863-014-0150-3) contains supplementary material, which is available to authorized users.

## Background

Lentil (*Lens culinaris* Medik.) is a self-pollinating, diploid (2n = 2× = 14) grain legume crop with a large genome size (c. 4 Gbp) [1]. It is an important source of protein and fibre in the human diet, as well as being highly valuable as feed and fodder for livestock. Moreover, lentil plays an important role in crop rotations due to its capacity to fix atmospheric nitrogen [2,3]. Contemporary lentil has been inferred to be the product of a single domestication event [4], associated with the Neolithic Agricultural Revolution which is thought to have taken place around 7000 BC in the Eastern Mediterranean [5]. Cultivation then spread rapidly to the Nile Valley, Europe and Central Asia [6,7], followed by Pakistan, India and South America. Subsequently, introductions were made to cultivation zones in the New World (Mexico, Canada, USA and Australia) [8]. Lentil is currently grown widely throughout the Indian sub-continent, the Middle East, northern Africa, southern Europe, North and South America, Australia and western Asia [9-11]. World production of lentil is estimated at 4.4 million metric tonnes from an estimated 4.2 million hectares, with an average yield of 950 kg/ha [12].

Numerous landraces of lentil have been sampled from different geographical regions world-wide, and are now preserved within the Australian Grains Genebank (AGG), Horsham, Victoria, Australia. Many of these landraces are yet to be exploited for breeding activities. The key to increases in lentil yield is the conservation and surveillance of existing genetic diversity for broadening the use of available genetics [13]. One primary objective of germplasm conservation is to assess, maintain and catalogue available genetic variation within and between landraces in order to support their use in breeding programs. Genetic diversity between parental genotypes in crossing programs has been demonstrated to be important for effective genetic gain [14].

Genetic diversity in both cultivated and wild lentil has been explored using several approaches, including morphological and physiological markers, isoenzymes, DNA-based markers such as randomly amplified polymorphic DNAs (RAPDs), inter-simple sequence repeats (ISSRs) and amplified fragment length polymorphisms (AFLPs) [3,7,11,15-17]. Morphophysiological markers have been commonly used as a first step in germplasm characterisation, but the time required for processing of candidate accessions is significant. Analysis of quantitative trait variation can also provide an indication of genetic diversity present within a population, and such methods have been successfully used to measure phenotypic diversity in germplasm collections for a variety of crops including lentil [18,19]. However, DNA-based markers provide the most versatile systems for diversity studies. Genetic variation within southern Asian lentil germplasm was studied using RAPD markers, and the lowest diversity was detected in germplasm obtained from Pakistan, Afghanistan and Nepal [7]. Both RAPD and ISSR markers were used to explore genetic diversity in a collection of Italian landraces, and the authors demonstrated the advantages of the latter over the former for discrimination of closely related genotypes [20]. Characterisation of genetic diversity and population structure of Ethiopian lentil landraces was also performed using ISSRs, and recommendations were made for germplasm conservation and breeding programs [11].

A number of studies have reported the use of SSR markers for germplasm characterisation in a multiplicity of crop species [21,22], but due to limited availability, the use of such systems has been restricted for lentil cultivars. As a consequence of recent advances in sequencing and genotyping technologies, it has become possible to develop genomic resources for relatively understudied crop species such as lentil at an acceptable cost. Recently, a number of transcriptome studies for lentil have generated expressed sequence tag (EST) databases, and a large number of EST-derived SSRs and SNPs have been made available [23,24]. Both SSR and SNP markers are reliable and co-dominant in nature. However, operational challenges in the use of SSRs have arisen due to a number of problems. Accurate allele sizing is difficult, because of PCR and electrophoresis artefacts; PCR competition effects can cause unequal allele amplification, which results in an inability to observe heterozygotes; amplification based on secondary priming sites may occur; and null alleles may arise from mutations in the primer region flanking the SSR [25,26]. As a consequence, SNPs offer an attractive alternative, due to their high abundance within the genome, suitability for use in high-multiplex ratio for high-throughput genotyping, and capacity for automated analysis. In addition, SNP discovery from transcribed regions of the genome provides the basis for establishment of a direct link between sequence polymorphism and putative functional variation [27].

Assessment of genetic diversity in lentil is desirable for prospective future breeding activities, in terms of broadening and maintaining the diversity of the genetic base, improving opportunities for selection of improved genetics and cultivar identification. In the present study, the genetic diversity of 505 lentil cultivars and landraces obtained from different geographical regions and preserved within the AGG has been determined through the use of a genotyping tool based on 384 SNP markers.

## Methods

### Plant materials and DNA extraction

A total of 505 accessions of lentil (*Lens culinaris* Medik.), including cultivars (111) and landraces (394), were obtained from the AGG, Horsham, Victoria, Australia. All available passport data from these accessions is summarised in Additional file 1. Young leaf tissue from one field-grown plant per accession was harvested and stored immediately in 96-well microtube plates. Total genomic DNA was isolated after grinding (MM 300 Mixer Mill system, Retsch., Germany) using the DNeasy 96 plant mini kit (QIAGEN, Germany). DNA was suspended in 1 × TE buffer and further diluted to approximately 50 ng/μl prior to SNP genotyping.

### SNP genotyping

A sub-set of 384 SNP markers was assayed across all plant samples (Additional file 2). These SNPs were chosen on the basis of informative data from previous SNP discovery and linkage mapping experiments (data not shown). All of these SNPs met the assay design criteria of possessing sufficient 5’- and 3’- flanking sequence information and absence of other known SNPs in their vicinity. A designability score, as calculated for each SNP by Illumina (San Diego, CA, USA), that was higher than 0.6 predicted high rates of assay conversion. A total of 250 ng of genomic DNA from each genotype was used for locus-specific amplification, after which PCR products were hybridised to bead chips via the address sequence for GoldenGate assay detection on an Illumina iSCAN Reader. On the basis of obtained fluorescence, allele call data were viewed graphically as a scatter plot for each marker assayed using GenomeStudio software v2011.1 with a GeneCall threshold of 0.20.

### Genetic diversity and population structure analysis

The genetic structure of the germplasm collection was first analysed by performing PCoA implemented in the program GenAlex 6.41. Basic statistics were calculated using the genetic analysis package PowerMarker (ver. 3.23; [28]) for diversity metrics at each locus, including the total number of alleles (N_A_), allele frequency, minor allele frequency, heterozygosity, gene diversity (GD), and polymorphism information content (PIC). Genetic similarities between each pair of accessions were measured by using an in-house customised program Genomic Relationship Matrix (genomicRelMatp). A heat map was generated using the R package. The NJ dendrogram from cultivar data was generated using the DARwin package based on genetic distance calculated using NTSYS v2.1.

For analysis of population structure, a Bayesian model-based analysis was performed with STRUCTURE v2.3.4 [29]. The posterior probabilities were estimated using the Markov Chain Monte Carlo (MCMC) method. The MCMC chains were run with a 20,000 burn-in period, followed by 20,000 iterations using a model allowing for admixture and correlated allele frequencies. At least 20 runs of STRUCTURE were performed by setting K from 1 to 15, and an average likelihood value, L (K), across all runs was calculated for each K (L(K) = an average of 20 values of LnP(D)). The admixture model was applied and no prior population information was used. The log-probability of the data, given for each value of K, was calculated and compared across the range of K [30].

## Results

### SNP polymorphism

A sub-set of 384 genome-wide distributed SNPs was used to assess genetic diversity within lentil germplasm, of which 192 were assigned to locations on the lentil genetic map (Table 1). Of the 384 SNPs, 274 (71.3%) obtained successful amplification and detected polymorphism, while of the remaining 110 SNPs, 90 either failed to amplify or produced inconsistent results and 20 were monomorphic in majority of the genotypes (>99%). This sub-set of 274 SNPs was further filtered for percentage of missing data, and any SNP loci with more than 40% of missing data were excluded from further analysis in order to generate a final set of 266 loci (of which 147 were assigned to the lentil genetic map). All of the 505 genotypes included in this analysis exhibited < 40% missing data individually. SNP loci were categorised in terms of the numbers of alleles, gene diversity, and PIC value. Gene diversity and PIC values varied from 0.108 (SNP_20002225) to 0.500 (SNP_20000915) and from 0.102 (SNP_20002225) to 0.375 (SNP_20000915), with averages of 0.419 and 0.328, respectively. The minor allele frequencies (MAF) per locus varied from 0.501 (SNP_20000915) to 0.943 (SNP_20002225) with an average of 0.673, with only 5 SNPs showing MAF > 0.90. Heterozygosity was lowest at loci SNP_20002225 and SNP_20001463 (both at 0.036), followed by SNP_20001223 (0.043) and SNP 20005402 (0.046) (Additional file 3).

**Table 1:** **Number of SNP markers used from different linkage groups of lentil**

**Linkage group**	**No of SNPs used**
Lc1	47
Lc2	24
Lc3	20
Lc4	44
Lc5	30
Lc6	26
Lc7	1
Unmapped	192

### Genetic diversity analysis

In the first instance, the genetic similarity between studied genotypes was quantified using a genomic relationship matrix (Additional file 4) and a heat map was generated after sorting of data on the basis of country-of-origin. Approximately 10 clusters of significant size were obtained, in most cases leading to grouping of genotypes from the same country-of-origin (unpublished data). However, the heat map data was not sufficient to provide conclusions on the genetic relationships between different accessions, due to the large number used in the study, as well as the low level of diversity that is present in general within lentil gene pool. Therefore, in order to further understand the genetic relationships between lentil genotypes for breeding purposes, data from commercial cultivars and landraces were analysed separately. Based on the calculation of genetic distance between 111 cultivars, the most divergent pair were Indianhead and Northfield (Nei’s coefficient value 0.210937; Additional file 5, sheet 1) while the landraces, ILL0166 and ILL5062 exhibited maximum genetic distance (Nei’s coefficient value 0.23148, Additional file 5, sheet 2). Two USA lentil cultivars LC05600043T and Palouse were genetically most similar (Nei’s coefficient value 0.0027715; Additional file 5, sheet 1) and similarly the genetic distance between two landraces ILL0369 and ILL0373, both originated from Chile, was the smallest (Nei’s coefficient value 0.003244; Additional file 5, sheet 2).

**Figure 1:**
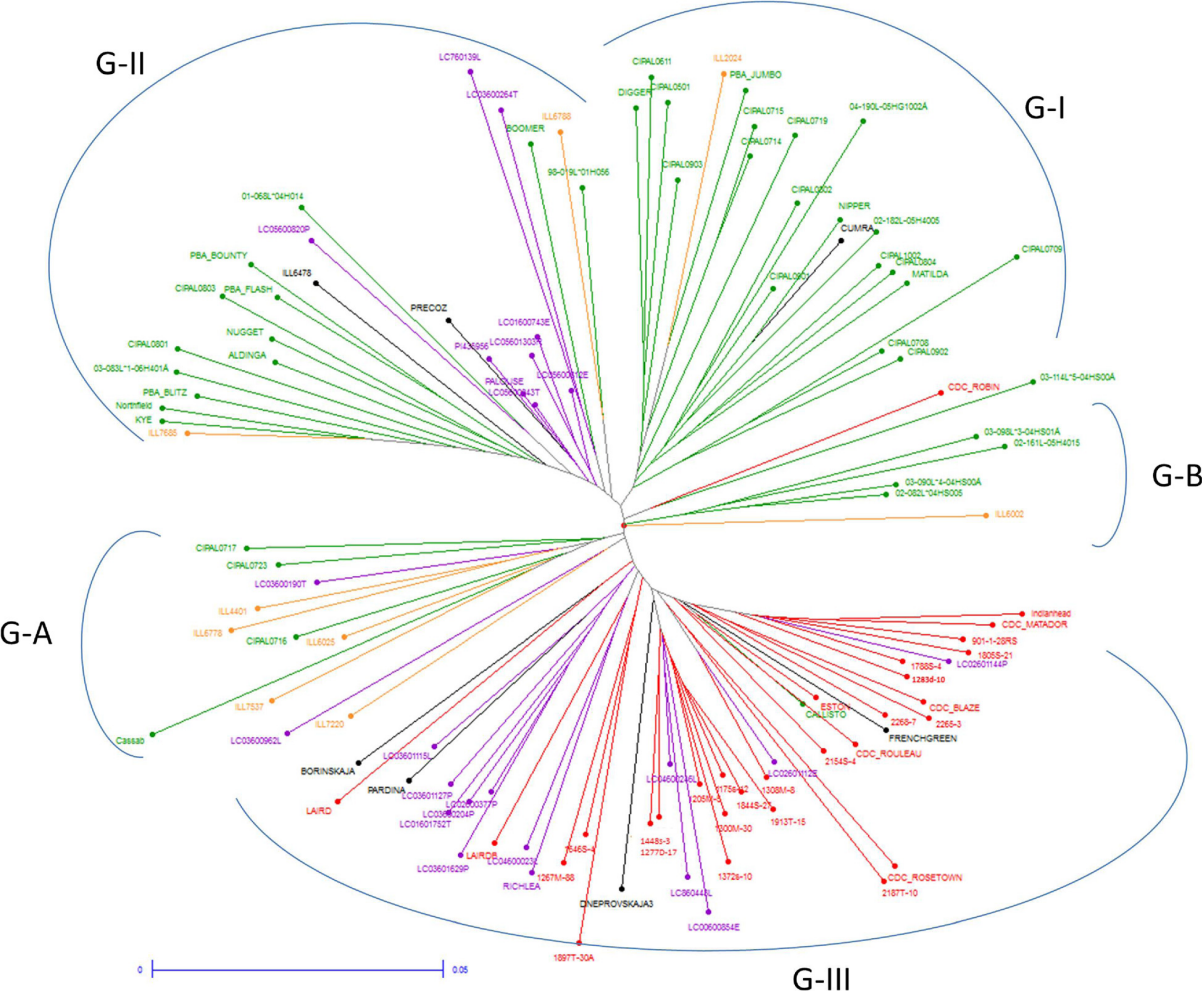
**NJ dendrogram generated based on genetic distance calculation from NTSYS v2.1.** Australian cultivars are shown in green (G-I, G-II), Canadian in red (G-III), USA in purple (G-I and III), breeding lines from ICARDA in orange.

A NJ dendrogram of commercial cultivars was generated (Figure 1). All lentil cultivars were assigned to three major groups (G-I, G-II and G-III) and two small outgroups (G-A and G-B). Group I mainly consisted of Australian cultivars, Group II was mainly composed of cultivars from Australia with some USA cultivars, and most of the cultivars from USA and Canada were assembled into Group III. The two small outgroups (G-A and G-B) were composed of Australian lentil cultivars with some breeding lines from the International Centre for Agricultural Research in the Dry Areas (ICARDA) (ILL4401, ILL6778, ILL6025, ILL7537 and ILL7220).

### Population structure analysis

**Figure 2:**
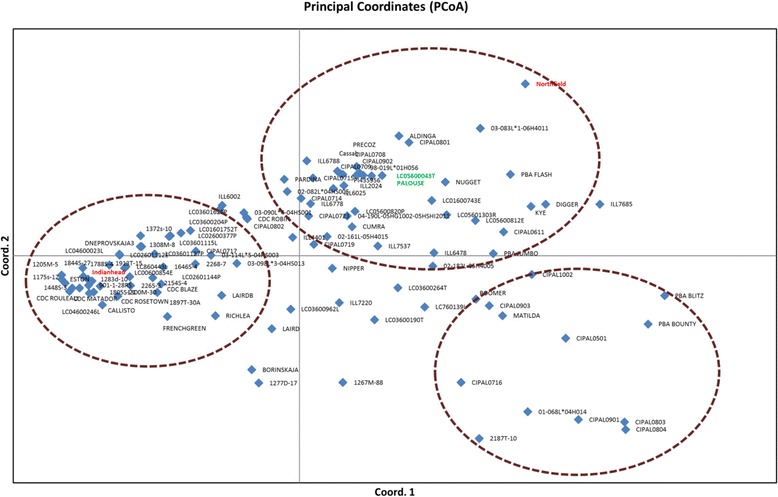
**Principal coordinate analysis (PCoA) plot generated from genetic distance calculations using the GENALEX package for 111 lentil cultivars.**

**Figure 3:**
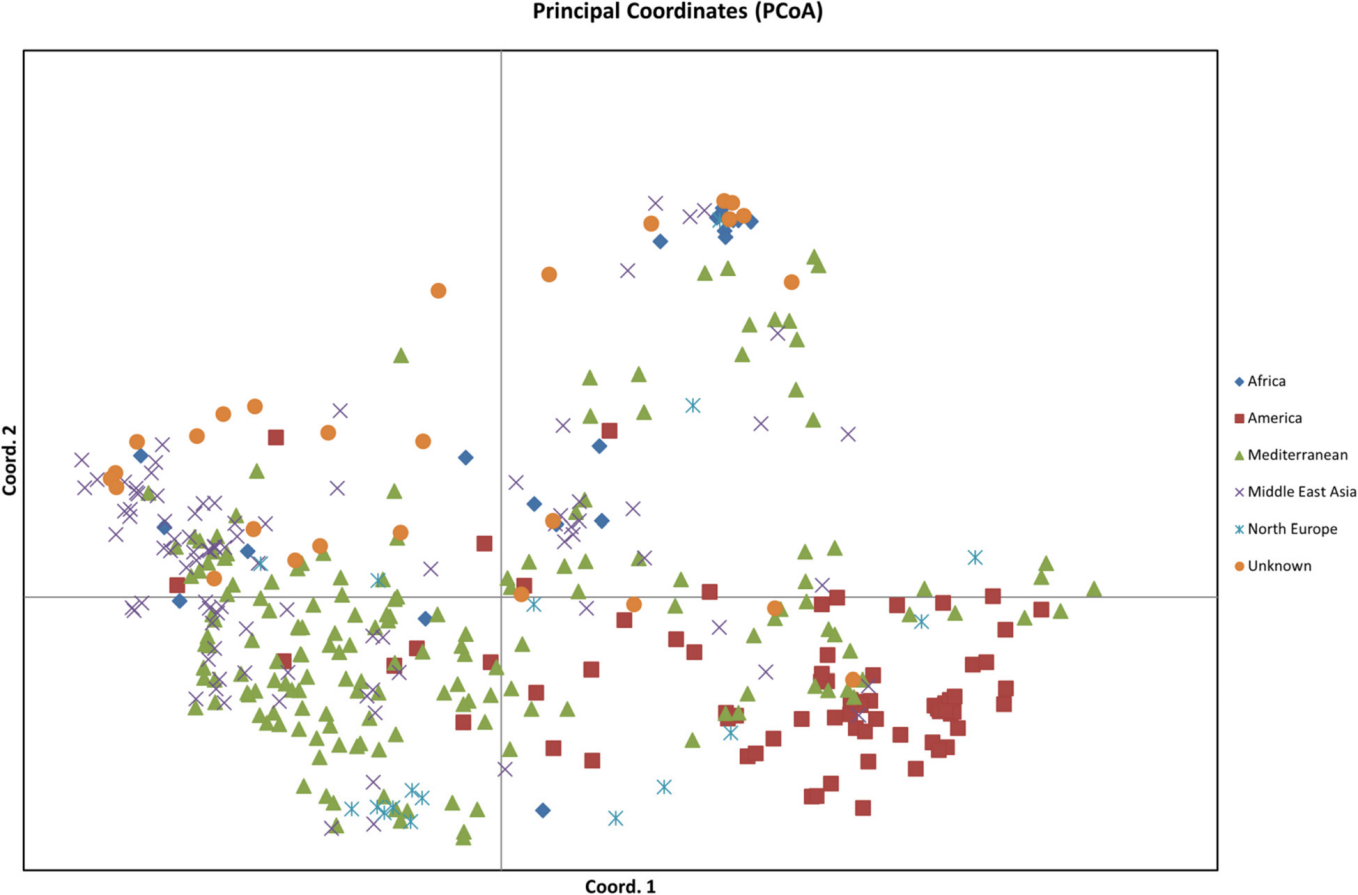
**Principal coordinate analysis (PCoA) plot generated from genetic distance calculations using the GENALEX package for 394 lentil landraces.** Different coloured labels indicate distinct geographical origins.

The genetic structure of the germplasm collection was analysed separately for both cultivars and landraces using PCoA and STRUCTURE. The PCoA of genetic distance between genotypes, based on SNP allele frequencies revealed an obvious differentiation between lentil genotypes. For cultivars, the first and second axes explained 37.16% and 18.30% of the total variance, and separated lentil cultivars into different clusters mainly based on geographical origin (Figure 2). Three major clusters were identified; cluster 1 containing most of the Canadian and USA-derived cultivars, while cluster 2 contained the majority of Australian cultivars along with some cultivars from USA, and cluster 3 was mainly composed of Australian cultivars. A different pattern was observed for landraces originating from c. 45 countries (Figure 3). The first and second axes explained 29.23% and 25.46% of the total variance, and separated landraces into different clusters. However, a weak correlation between geographical origin and clustering was observed. Consequently, landraces from various countries were grouped according to a larger geographical zone for interpretation of the data. For example, landraces from Chile, Peru, Mexico, Argentina, Colombia, Guatemala and Ecuador were categorised as American accessions, while landraces from Turkey, Greece, Syria, Tunisia, Spain, Morocco, Italy, Lebanon, Egypt, Cyprus and Algeria were classified as Mediterranean in origin. Most of the landraces from America, Africa, Northern Europe and Middle-East Asia were incorporated into these general groups. However, Mediterranean landraces, chiefly from Greece and Turkey, were dispersed across the PCoA plot (Figure 3).

**Figure 4:**
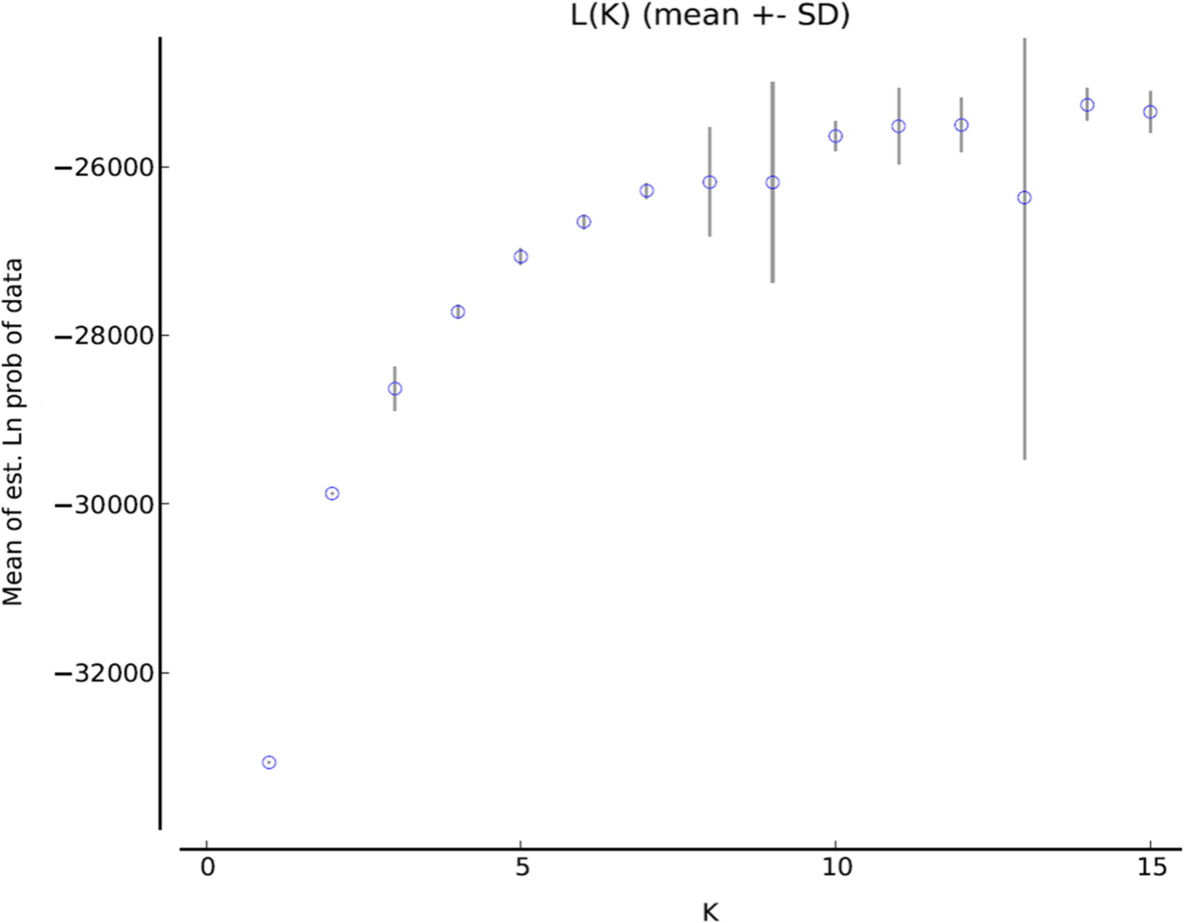
**Estimated number of clusters obtained for lentil cultivars with STRUCTURE for K values from 1 to 15 using SNP data.** Graphical representation of estimated mean L(K) values showing the clustering of different cultivars.

**Figure 5:**
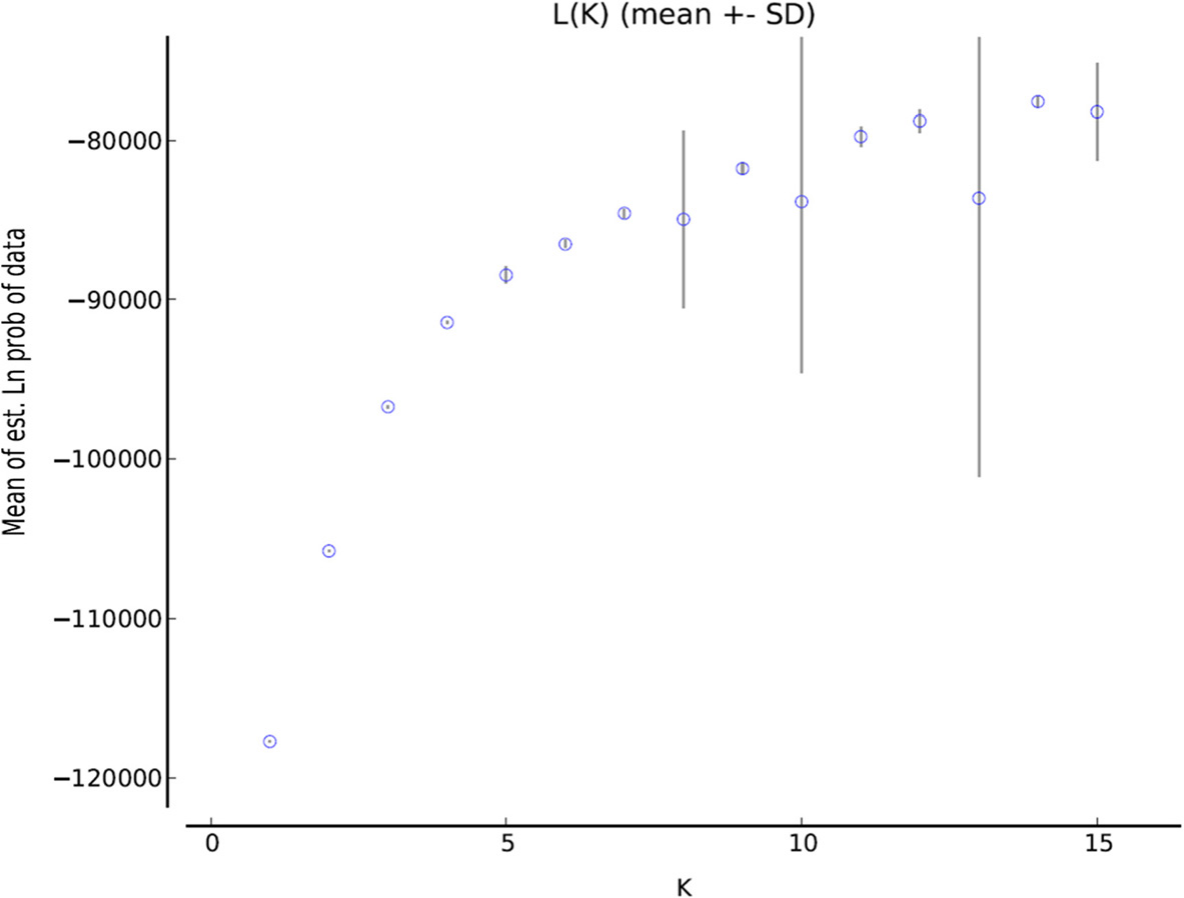
**Estimated number of clusters obtained for lentil landraces with STRUCTURE for K values from 1 to 15 using SNP data.** Graphical representation of estimated mean L(K) values showing the clustering of different landraces.

The SNP datasets were further used for the model-based Bayesian clustering method as implemented in STRUCTURE. The log likelihood of each K was calculated as L(K). The estimation of true value of K was based on the observation that L(K) reached a plateau (or continued to increase slightly) and displayed high variance between runs. This analysis showed an optimum value of K = 3 for commercial cultivars (Figure 4) and K = 5 for landraces (Figure 5). The outcomes of the analysis coincided with the three distinct clusters identified for commercial cultivars from the genetic diversity analysis. However, the value of K = 5 for landraces proved too complicated to allow assignment of a population structure to the whole set based on geographical origin. An attempt was made to categorise the landraces based on climatic data, however, this was not helpful for further resolution of the results (unpublished data).

## Discussion

### Suitability of SNP markers for germplasm characterisation

Recent advances in marker technologies have enabled the routine use of high-throughput, low-cost markers for germplasm characterisation and to select for favourable alleles in plant breeding programs. SNP markers offer an ideal marker system that is highly polymorphic, co-dominant, accurate, reproducible, high-throughput, low-cost and highly informative. In the present study, the suitability of a 384-plex SNP GoldenGate assay tool has been demonstrated for genotyping of a lentil genetic resource collection. Despite broad diversity within the germplasm collection due to inclusion of landraces from multiple countries-of-origin, the majority of SNP markers were efficiently detected. The success rate (69.2%) was slightly lower than that obtained for other crops such as soybean (89%; [31]), field pea (91%; [32]) and grape (92%; [33]), based on the same genotyping technology. This effect may be due to the lower levels of genetic diversity that are present within the set of lentil accessions assessed in the current study, in comparison to the other species. The average SNP frequency between two genotypes has been reported to be 0.21 per kb (*L. culinaris*) and 0.31 per kb (*L. ervoides*) [24]. These values are lower than for other related legume species such as soybean (2.7 SNPs per kb; [34]), field pea (2.7 SNPs per kb; [35]) and *Medicago truncatula* Gaertn. (1.96 SNPs per kb; [36]), supporting the view that lentil germplasm is relatively similar in nature.

Information content of markers was assessed on the basis of a number of different criteria, the most fundamental being number of alleles, higher values of which are likely to lead to higher polymorphisms in any given germplasm set. However, this criterion is most relevant to SSR markers, which are capable of displaying multiallelic structure. For SNPs, in contrast, for which biallelic patterns are standard (using Goldengate assays), expected heterozygosity is a more accurate measure of polymorphism as this parameter measures distribution of alleles across the germplasm under examination. In general, the level of genetic diversity quantified as heterozygosity based on SNP markers was approximately half that estimated through use of SSR markers [33]. This potential disadvantage of SNP-based systems may be overcome either through use of a large number of markers, or by considering haplotypic structure for each locus, instead of individual SNP loci. The differences between SNPs and SSRs in terms of levels of genetic diversity result from the mutational properties of these two marker types. Minor allele frequency is a measure often used to assess information content for SNP loci, and is related to expected heterozygosity. For all SNPs, an average expected heterozygosity value of 0.15 was identified, identical to that obtained from other studies [26,33,37].

### Assessment of genetic diversity and population structure

Estimation of the degree of differentiation between accessions that are included in a crossing program is useful for selection of parental genotypes. The maximum distance (Figure 1) was calculated between Indianhead, a Canadian cultivar (G-III), and Northfield, an Australian cultivar bred in Syria (syn. ILL5588) (G-II), which are derived from highly separated localities and breeding populations. Conversely, two cultivars from the USA (Palouse and LC05600043T) were most genetically similar among all cultivars studied (Figure 2, G-II). The USA-derived lentil cultivars were genetically closer to those from Canada than Australia, also supporting these observations. Some counter-examples in which cultivars from different geographical origins grouped together were also observed. For example, a single French cultivar (French Green) clustered with cultivars from Canada and USA.

Based on knowledge of the pedigrees of Australian cultivars and breeding lines that were included in this study, the PCoA obtained a number of consistent relationships. For example, variety Nipper, which was derived from a three-way cross between Indianhead and (twice-over) Northfield, is located mid-way between these two lines. Similarly, CIPAL0715 and CIPAL0714 (released as variety Grampians), lie between the parental lines Nugget and French Green, while the widely adopted variety PBA Flash is positioned close to mid-way between its parents, Nugget and ILL7685. Many of the breeding lines in the largest cluster of Australian varieties (02-161 L-05H4015, 02-182 L-05H4005, and 04-190 L-05HG1002-05HSHI2011) were located at intermediate positions between their parents.

In contrast, some other varieties appear in positions inconsistent with recorded ancestry. For instance, CIPAL0801 (syn. PBA Bolt) appears close to Aldinga, distant from the parents, ILL7685, Nugget, and Matador. In the same way, Boomer and CIPAL0501 are sister progeny of a Digger × Palouse cross, but are located in a separate cluster. Anomalous placements were also observed for PBA Blitz, PBA Bounty, CIPAL0803 (syn. PBA Ace), 01-068 L-04H014 and CIPAL0901, all being elite Australian cultivars or breeding lines. The explanation of such anomalous results is not clear, although errors in pedigree record-keeping, or labelling of seed samples, are more probable. In general, however, PCoA confirmed many of the known relationships between cultivars. Given the relatively short history of Australian lentil breeding, such affinities may be attributable to the contributions of original source germplasm, predominately landraces or cultivars obtained from ICARDA.

The analysis of the landraces did not reveal a strong correlation between geographical origin and genetic diversity. It is generally accepted that landraces may consist of highly diverse mixtures of different genotypes, and may hence require substantial within-accession sampling for a meaningful analysis of genomic diversity [17]. The landraces originating from Mediterranean regions, especially those derived from Turkey and Greece, were highly diverse from one another, suggesting that a substantial level of genetic variation is presented within this class of germplasm. This effect could be related to the domestication of lentil, which is known to have occurred in the eastern Mediterranean [5], in which non-domesticated *Lens* species are endemic. Following the initial domestication event, cultivation of lentil spread to Europe, Central Asia, Pakistan, India and South America [8], and the narrower genetic base and clustering between accessions from these regions is consistent with a history of limited introductions. A number of studies have revealed that lentil germplasm from the Mediterranean region is characterised by higher genetic diversity than those of the USA and Asia [17,38-40].

Knowledge of genetic variation and genetic relationships between lentil landraces is important for efficient germplasm preservation, characterisation and subsequent use by lentil breeders. The narrow genetic base of the cultivars compared to the landraces, as shown by genetic distance estimates, reveals a relatively untapped pool of genetic diversity that could be highly valuable for further advances in yield potential, along with resistance to biotic and abiotic stresses. In addition to this, information on regional differentiation has practical significance for the management of germplasm and to assist selection of parental genotypes for breeding activities. Selection of genetically diverse landraces as parents should contribute to genetic gain through identification of superior progeny combinations from within breeding populations. This will also lead to cultivars with superior local adaptation, if the populations are evaluated and analysed carefully. Furthermore, information on regional differentiation should provide evidence for identification of parents with enhanced abiotic stress tolerance or resistance to biotic challenges.

The indicative number of clusters obtained from use of STRUCTURE was K = 5, but substantial overlaps were observed between different clusters. The result of the present study for lentil accessions, revealing limited correspondence between geographical origin and genetic diversity, is similar to that obtained in previous studies of other crops such as field pea [41] and safflower [42]. This phenomenon suggests that selection of parents in breeding programs should be made on the basis of systematic assessment of genetic distance between base populations, rather than geographical difference. Such divergence between parental genotypes is likely to reflect accumulated allelic differences [14], including those at target agronomic loci, allowing maximised potential for selection of desirable traits or to introgress favourable gene variants in backcross-based programs. Once again, this process should lead to the development of superior locally adapted cultivars.

### Applicability of SNP diversity data for genome-wide association studies

Of the 384 SNPs used in the current study, genetic map positions were known for 192 (50%). Such information could contribute to future detailed genome wide-association studies (GWAS) studies [43] for lentil. The number of SNP markers required for effective GWAS is a function of average extent of linkage disequilibrium (LD) within the relevant genome. The limited genetic diversity and inbreeding reproductive habit of lentil will probably lead to extensive LD, and hence a lower marker requirement than for outbreeding species with high levels of genetic diversity, such as grasses [44,45] and oilseeds [46]. Despite these favourable properties, the 384-plex SNP genotyping tool described here is unlikely to be insufficient for GWAS in isolation. Nonetheless, an enhanced version of multiplexed SNP genotypic analysis, in concert with the detailed knowledge of population diversity and stratification as described in the present study, will provide a basis for any future GWAS studies for lentil.

## Conclusions

Assessment of genetic variation among a global collection of lentil cultivars and landraces was performed using a set of genome-wide distributed SNP markers. Genetic diversity analysis revealed clear grouping within cultivars based on geographical origin, but no such correspondence was observed within landraces collection. This result indicates that assessment of genetic diversity is critical for choice of germplasm suitable for breeding activities, and the data presented in the present study will highly assist such efforts.

## Additional files

Additional file 1:
**Passport data for 505 lentil accessions used in the current study.** This file contains all passport data available for 111 lentil cultivars and 394 landraces used in the current study, indicating geographical origin, source, accessibility, taxonomy, description and pedigree. Additional file 2:
**Details of 384-plex SNP-OPA design: This file contains names and sequence information for all SNP markers used in the genetic diversity study.**
Additional file 3:
**Basic statistics data obtained from SNPs used in the current study using PowerMarker.** This file contains all information on basic statistics for SNP loci including minor allele frequency, gene diversity, PIC value and heterozygosity value. Additional file 4:
**Genetic similarity matrix calculated from 505 lentil accessions using Genome relationship matrix pipeline.** This file contains the genetic similarity indices between different lentil cultivars and landraces. Additional file 5:
**Nei’s coefficient data from lentil cultivars and landraces.** This file contains genetic distance data between lentil cultivars (sheet 1) and landraces (sheet 2) calculated using NTSYS software.
